# Clinical Characteristics and efficacy of chemotherapy in sclerosing epithelioid fibrosarcoma

**DOI:** 10.1007/s12032-018-1192-6

**Published:** 2018-09-05

**Authors:** Winston Chew, Charlotte Benson, Khin Thway, Andrew Hayes, Aisha Miah, Shane Zaidi, Alex T. J. Lee, Christina Messiou, Cyril Fisher, Winette T. van der Graaf, Robin L. Jones

**Affiliations:** 1 Sarcoma Unit, Royal Marsden Hospital, Fulham Road, London, SW3 6JJ UK; 2 Division of Molecular Pathology, Institute of Cancer Research, London, UK; 3 Division of Radiotherapy and Imaging, Institute of Cancer Research, London, UK; 4 Division of Clinical Studies, Institute of Cancer Research, London, UK

**Keywords:** Sclerosing epithelioid fibrosarcoma, Treatment, Prognosis, Chemotherapy

## Abstract

**Background:**

Sclerosing epithelioid fibrosarcoma (SEF) is a very rare soft tissue sarcoma subtype. Clinically it is an aggressive tumour; however, to our knowledge there are no published reports regarding the efficacy of chemotherapy in SEF. Therefore, the aim of this study was to document the outcome of a series of patients with SEF treated at a single referral centre with reference to systemic therapy.

**Methods:**

A retrospective search of a prospectively maintained database was performed to identify all patients diagnosed with SEF between 1990 and 2017. The diagnosis was confirmed in each case by a dedicated soft tissue sarcoma pathologist. We analysed those with recurrent disease and the effect of systemic chemotherapy in the metastatic setting.

**Results:**

Thirteen patients were identified, median overall survival from diagnosis and metastasis were 47.3 (95% CI 25.0–131.9) and 16.3 (95% CI 5.3–20.6) months, respectively. In total, 12 (92.3%) patients developed metastatic disease of which 10 died of disease, 1 was lost to follow-up and 1 had recently commenced palliative treatment. Among the 10 patients with metastatic disease, 7 received palliative chemotherapy. Palliative chemotherapy resulted in partial response in 1 patient, stable disease in 3 patients and progressive disease in 3 patients. Median time to disease progression was 2.7 (95% CI 1.2–4.4) months. Two of 13 patients were treated with adjuvant chemotherapy, receiving 6 cycles of liposomal doxorubicin and 1 cycle of doxorubicin, respectively, with a metastasis-free survival of 28.2 and 7.1 months, respectively.

**Conclusion:**

SEF is an aggressive sarcoma subtype with a poor outcome and with limited responsiveness to conventional chemotherapy. Patients with this subtype should be considered for participation in clinical trials with novel agents. Further investigation into the biology of this rare disease is required to improve outcomes.

## Introduction

Sclerosing epithelioid fibrosarcoma (SEF) is a rare and aggressive sarcoma subtype with little more than a hundred cases described in the literature. SEF was first described by Meis-Kindblom et al. in 1995 [1] as a proliferation of neoplastic oval or round cells arranged in cords or a nest-like distribution against a collagen background with prominent features of sclerosis. This variant of fibrosarcoma has a typically low-grade histological appearance with a low mitotic count and lacks the presence of necrosis. When present, the carcinoma-like infiltrating growth pattern in SEF differentiates this malignancy from other epithelioid tumours. Alternatively, when these features are absent, the deceptively bland morphology compounds the difficulty in distinguishing fibrosarcoma from other fibrous growths (i.e. desmoid tumours, nodular fasciitis). SEF typically originates in the lower extremities with a high propensity for local recurrence after surgical resection and eventual metastasis to the lungs and bones [1]. The rarity of this disease [2] and the challenges of establishing a positive histological diagnosis have resulted in limitations to clinical and biological understanding of SEF.

However, there have been improvements in identifying SEF and distinguishing it from other types of fibrosarcoma. Doyle et al. reported that the immunohistochemical expression of MUC4 was seen in 78% of SEF and when coupled together with *FUS* gene, rearrangement was a sensitive and relatively specific marker for SEF [3] while Arbajian et al. found that *EWSR1-CREB3L1* gene fusions were distinguishing features between SEF and the morphologically similar low-grade fibromyxoid sarcoma [4]. Furthermore, attempts have been made to characterise SEF originating from specific sites of the body, Leona et al. recently described a series of primary SEFs of bone [5].

As the diagnosis of SEF becomes better defined, the question of optimal therapeutic strategy can be more confidently addressed. However, reports of systemic treatment outcomes have been limited to a handful of case reports where patients were treated with either single agent doxorubicin or in combination with ifosfamide, methotrexate, or cisplatin [6]. The aim of our study was to record the outcome of a consecutive series of patients with SEF treated at a single referral centre with particular reference to systemic therapy.

## Methods

A retrospective search of a prospectively maintained institutional database was performed to identify patients with SEF at the Royal Marsden Hospital. All data collection and usage for this study were approved by the hospital review board. Our study identified patients who were diagnosed with SEF between 1990 and 2017, including those with a retrospective diagnosis based on a review of histology following the first description of SEF in 1995. Histology slides of each case of SEF diagnosis were reviewed by a dedicated soft tissue sarcoma pathologist (CF, KT) for the purpose of this study. Tumour grade was assessed using the FNCLCC system while tumour size was taken from the largest dimension recorded in the pathological report. Associated baseline clinicopathological data, treatment details and patient outcome data were collected by retrospective notes review. All patients were treated in a specialist sarcoma oncology clinic with treatment decisions based on multi-disciplinary team discussion and the discretion of the treating physician. Overall survival and time to metastasis were defined as the time of SEF diagnosis to death or metastasis, where SEF diagnosis was defined as the time at which that patient presented with symptoms caused by SEF. Additionally, systemic chemotherapy response was evaluated using RECIST 1.1, where time to disease progression was defined as time from 1st dose of chemotherapy to time of radiological disease progression. Response CT scans were performed as per routine institutional practice, generally after every two cycles of systemic therapy. Best response was defined at time of first response assessment CT. Overall survival was measured both from date of initial diagnosis and from date of first dose of first-line chemotherapy, until date of death or last clinical follow-up. Statistical analysis was calculated using Medcalc version 18.

## Results

### Patient clinicopathological characteristics

A summary of baseline clinicopathological characteristics is displayed in Table 1. In total, we identified 13 patients who were diagnosed with SEF between 1990 and 2017. SEF was found in balanced distribution among both sexes. Median age of diagnosis was 44.6 years old (26.5–65.6 years) with 3/13 (23.1%) patients diagnosed below the age of 35 years.

**Table 1:** Clinicopathological characteristic and management of each patient

ID	SEX	Age at diagnosis (years)	STATUS	Primary tumour site	Primary tumour size (cm)	Primary tumour grade	Metastasis-free survival (months)	Overall survival from diagnosis (months)	Number of radical resection (margin status)	Metastectomy	Total dose of adjuvant radiotherapy	1st line systemic chemotherapy (cycles)	Chemotherapy best response	Progression-free survival (months)	Overall survival from 1st line systemic chemotherapy (months)
1	F	65.5	DOD	Foot	3	1	18.7	24.9	2 (R1, R1)	No	NIL	Doxorubicin (2)	Stable	1.2	5.7
2	F	62.7	DOD	Thigh	Unknown	1	103.6	167.5	1 (R1)	No	NIL	Doxorubicin (4)	PD	2.3	4.6
3	F	27.1	DOD	Retroperitoneum pelvis	Unknown (but large)	3	Met @ diagnosis	7.3	0	No	NIL	Ifosfamide + doxorubicin (5)	PR	3.0	6.8
4	M	44.5	DOD	Buttock	25	1	5.3	112.7	1 (R1)	Yes	66 Gy	Doxorubicin (6)	Stable	7.0	28.4
5	M	37.8	DOD	Ant abdo	10	1	28.2	131.9	1 (R1)	Yes	NIL	Gemcitabine + docetaxel (2)	PD	1.2	40.4
6	F	41.4	DOD	Post chest	12	Unknown	7.1	52.6	1 (R1)	Yes	66 Gy	Ifosfamide + Doxorubicin (5)	PD	4.4	20.7
7	F	37.5	DOD	Head and neck	6	1	8.5	26.7	2 (R1, R1)	Yes	NIL	Ifosfamide + doxorubicin (6)	Stable	2.7	16.3
8	F	56.4	DOD	Buttock	9	2	Met @ diagnosis	11.1	0	No	NIL	–	–	–	–
9	M	64.8	DOD	Post chest	6	1	2.1	10.1	1 (R1)	No	NIL	–	–	–	–
10	F	74.5	DOD	Axilla	14	3	16.3	47.3	1 (R1)	No	50 Gy	–	–	–	–
11	M	37.1	AWD	Left posterior 11th rib	8	3	2.9	17.9	1 (R1)	No	NIL	–	–	–	–
12	M	56.3	LOFU	Retroperitoneum	Unknown	Unknown	20.6	21.6	2 (R1, R1)	No	NIL	–	–	–	–
13	M	26.5	ANED	Thigh	3	1	NED	105.0	2 (R1, R0)	–	–	–	–	–	–

The most common primary tumour sites were the lower limbs (7/13, 53.8%) followed by the chest (5/13, 38.5%) and the neck (1/13, 7.7%). Grade and maximal dimension of primary tumour were available for 11/13 and 10/13 patients, respectively—7/11(64%) grade 1, 1/11 (9%) grade 2 and 3/11 (23%) grade 3. Median primary tumour size was 9 cm (range 3–25 cm).

Eleven out of 13 (84.6%) patients had localised disease at first presentation, all of whom underwent treatment with radical intent. Local recurrence occurred in all 11 patients and ultimately 10/11 (90.9%) patients developed metachronous metastatic disease. In patients that developed metachronous metastatic disease, median metastasis-free interval following diagnosis was 12.4 months (95% CI 5.3–20.6). Two out of 13 (16.7%) patients presented with synchronous metastatic disease. The most common metastatic sites were lung (8/12, 66.7%) and bone (6/12, 50%), other metastatic sites included the liver, brain and abdomen seen in 1 patient each. At the point of data cut-off, 10/13 patients had died of metastatic disease, one patient had recently commenced palliative chemotherapy for metastatic disease, one patient with metastatic SEF was lost to follow-up after 21.6 months post-diagnosis and one patient remained disease-free after nearly 9 years of follow-up after a second radical resection of locally recurrent SEF originating from the thigh.

### Surgical and radiological management of disease

Oncological management of all 13 patients is summarised in Table 1. In all 11 patients who underwent radical resection for localised disease, microscopic involvement of resection margins (R1) was reported. Three out of 11 patients, all of whom had large (> 10 cm), proximally located tumours, received post-operative radiotherapy to the surgical bed, with a total dose of 68 Gy or 50 Gy delivered in 2/3 and 1/3 patients, respectively. All radically treated patients experienced tumour relapse. Seven out of 11 had metastatic disease at first relapse. Four out of 11 initially had local recurrence only, with resection of recurrence attempted in all 4 patients. Three out of 4 patients ultimately relapsed with metastatic disease while only 1 patient had no evidence of disease with a follow-up of 105 months from initial diagnosis—notably, this was the only patient where R0 resection was achieved. Patients 4, 5, 6 and 7 underwent metastectomy to remove SEF metastases to the lungs and lymph nodes.

#### Systemic chemotherapy in SEF

Among the 12 patients with metastatic SEF, 7 patients received at least one line of systemic chemotherapy, 2 patients were unfit for chemotherapy and received best supportive care, 1 patient declined chemotherapy, 1 patient with bone-only metastases received zoledronic acid and 1 patient was lost on follow-up due to transfer of care. Table 1 outlines 1st line systemic chemotherapy administered to 7 patients with metastatic SEF and associated outcomes. All patients had radiologically progressive disease prior to commencement of chemotherapy. Three patients were treated with single agent doxorubicin and received a median of 4 (range 2–6) cycles. Three patients were treated with combination doxorubicin and ifosfamide, receiving between 5 and 6 cycles. One patient was treated with 2 cycles of combination gemcitabine and docetaxel. One patient had a partial response to doxorubicin/ifosfamide chemotherapy, 3 had stable disease (all with doxorubicin monotherapy) and 3 had progressive disease.

Only patient 5 had additional lines of chemotherapy; following progression after 2 cycles of 1st line gemcitabine/docetaxel, this consisted of 6 cycles of oral cyclophosphamide and 3 cycles of trabectedin for 2nd and 3rd line, respectively. Best response with cyclophosphamide was stable disease with an associated progression-free survival of 9.9 months while progressive disease was seen after 2 cycles of trabectedin.

#### Survival analysis

**Fig. 1:**
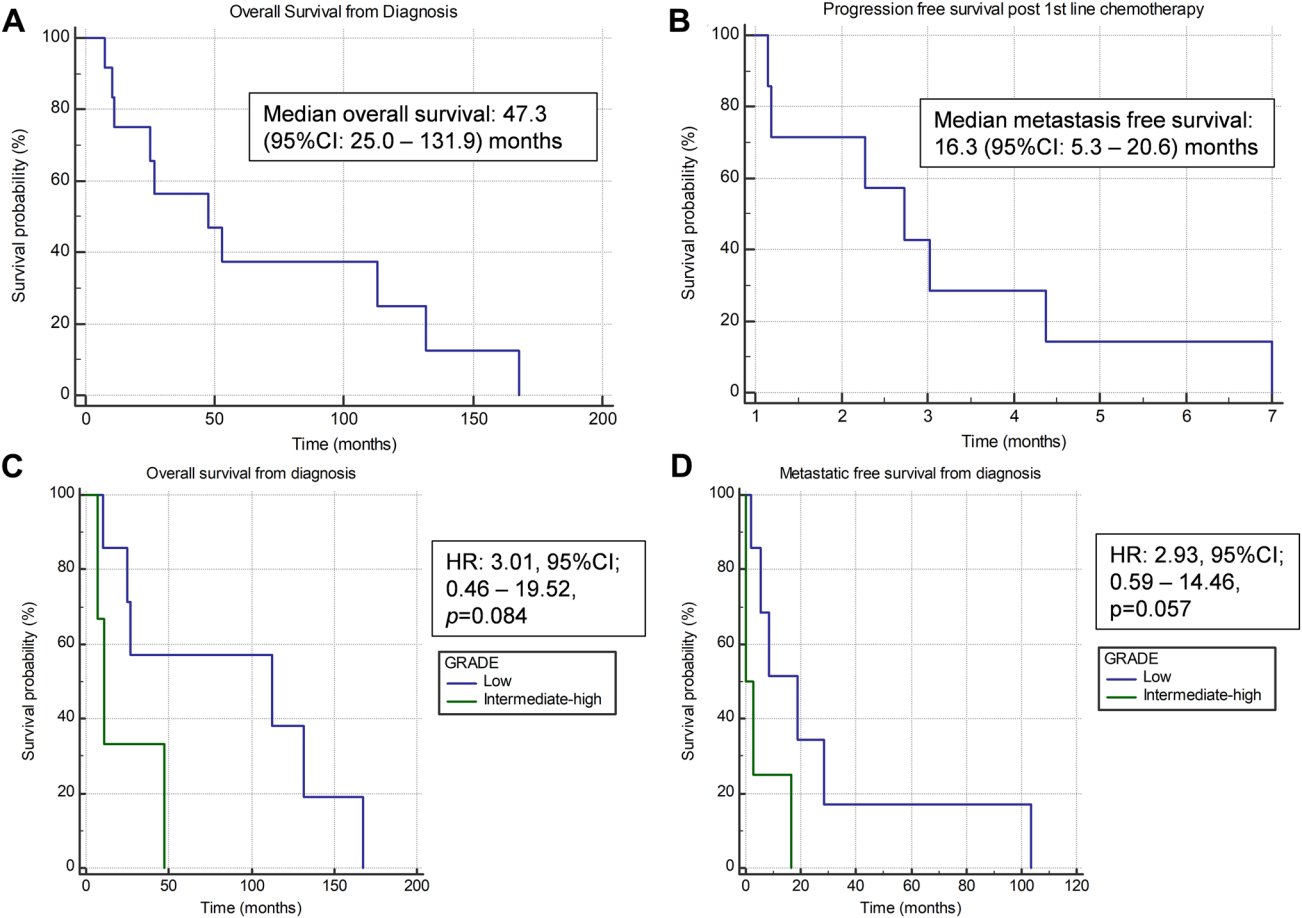
Kaplan–Meier curves for overall survival from diagnosis (**a**), progression-free survival from 1st line chemotherapy (**b**), overall survival from diagnosis based on primary tumour grade (**c**) and metastatic free survival based on primary tumour grade (**d**)

As illustrated by Fig. 1a–d and Table 1, median overall survival from diagnosis was 47.3 months (95% CI 25.0–131.9). Metastatic-free survival in patients initially diagnosed with primary disease was 16.3 months (95% CI 5.3–20.6). In patients that received chemotherapy, median progression-free survival post 1st line chemotherapy (Fig. 1b) was 2.7 months (95% CI 1.2–4.4). SEF that were intermediate/high grade at diagnosis had a non-significant trend to lower overall survival (HR 3.01, 95% CI 0.46–19.52, *p* = 0.084) and metastases-free survival (HR 2.93, 95% CI 0.59–14.46, *p* = 0.057). Primary tumour size and age of diagnosis were not significant predictors of overall and/or metastases-free survival.

## Discussion

Due largely to its rarity and having only relatively recently being recognised as a distinct diagnostic entity, SEF is associated with a limited published literature. Consequently, there are few published studies describing the clinical phenotype, molecular biology and optimal management of this cancer. We have reported the largest case series of SEF with annotated follow-up of treatment outcomes and have identified important features that should inform future management of a rare STS subtype that is associated with poor clinical outcomes.

In our series of 13 patients with confirmed SEF, a majority of patients initially presented with a primary tumour in the absence of demonstrable metastatic disease. The clinical epidemiology of SEF seen in our cohort is consistent with past reports by Meis-Kindblom et al. and Antonescu et al. [1, 7]. Specifically, SEF are capable of presenting in patients below 35 years old without gender preponderance and primary tumours were most commonly originating in the lower limbs or trunk. Additionally, while histologically primary SEF usually appear low grade, they exhibit a high risk of metastasis, typically to the lungs and bones. A possible explanation behind the cause of this apparent discrepancy between low tumour grade and metastatic potential, is the progression of malignancy in microscopic locally residual SEF. Kanno et al. described a case of SEF with local multiple recurrences over 12 years with eventual bone metastasis that had increased Ki67 expression over prior recurrence and the original primary tumour. Notably, the sole long-term survivor in our cohort was the only patient that achieved a R0 resection, re-emphasising the metastatic potential of microscopic locally residual SEF.

Anatomical site of primary tumour varied widely between individual patients and included proximal and distal extremities, trunk, head and neck and intra-cavity areas. Seven out of 13 patients had tumours with low histological grade yet did not demonstrate a significantly less aggressive clinical course compared to intermediate or high-grade tumours. While eventual progression to metastatic disease seems likely, our results suggest that low-grade tumours develop at a slower rate relative to intermediate or high-grade tumours.

Despite management in a specialist STS centre, microscopic involvement of surgical margins following radical resection was ubiquitous. This suggests a locally infiltrative disease process that may not be adequately captured by macroscopic or radiological assessment of tumour extent before or at the time of surgery. This should inform considerations of surgical approach and the use of pre- or post-operative radiotherapy in pursuit of optimal local disease control.

Among the 12 patients, only 3 received adjuvant radiotherapy, 2 of whom later experienced local recurrence, and all three developing metastases. Given the high rates of R1 resection and local recurrence, and an apparent mismatch between histological grade and clinical phenotype, indication for delivery of adjuvant radiotherapy in SEF may extend beyond general recommendations for STS.

In our series, 7 patients received at least one line of systemic therapy for advanced disease. Objective tumour response was seen in a single patient with a histologically high-grade tumour, but was associated with short duration of response. Disease stabilisation was seen in three patients treated with doxorubicin, with radiological disease control lasting 1.2, 2.7 and 7 months, respectively. Progressive disease was seen in the other patients after an assortment of regimens commonly used in the treatment of advanced STS. These findings suggest that SEF has a chemo-sensitivity in keeping with a large proportion of other STS subtypes, where occasional tumour sensitivity is demonstrated in the context of likely limited benefit delivered to a majority of patients. In terms of overall systemic chemotherapy response to SEF, similar median progression-free survival and overall survival were observed in systemic chemotherapy for other types of metastatic soft tissue sarcoma [8]. While, at present, it appears reasonable to continue to offer available regimens for advanced SEF, this must be done with anticipation of benefitting a minority of patients. Meanwhile, more effective and well-tolerated systemic therapies are in acute need. Thus, clinicians should consider offering patients participation in clinical trials [9]. Additionally, further investigation into potential therapeutic targets may provide more effective treatments. Arbajian et al. recently characterised a series of SEF and low-grade fibromyxoid sarcoma and compared gene expression against those expressed by fibroblast cells transfected with *EWSR1-CREB3L1*. They reported that additional genetic mutations may explain the differences in malignant potential between the two tumours. Specifically, SEF had recurrent *DMD* microdeletions and upregulation of CD24 and provided a possible lead for therapeutic targets [10].

Patients that were metastatic at diagnosis tended to be those with large deep-seated tumours located centrally [11]. The asymptomatic nature of SEF coupled with the large primary tumour size suggested a long-standing malignancy and is in keeping with the possibility of SEF slowly developing the ability to metastasize overtime in these patients.

While SEF had an extremely high potential to metastasize, there is a marked heterogeneity in the aggressiveness of the tumour. Nearly half of our patients reached the 3-year survival mark with some surviving for more than 10 years while conversely a third passed away within the first year. Additionally, even when metastasis has occurred, a handful of patients continue to be clinically well for a number of years before having to start systemic chemotherapy. This heterogeneity in the clinical phenotype of SEF may, in part, be related to variation in underlying molecular pathology. Doyle et al. [3] described two different subsets of SEF. Firstly, a MUC4-positive SEF that lack *FUS* rearrangement which may be related to the less malignant low-grade fibromyxoid sarcoma. Secondly, a SEF lacking MUC4 expression that could be a more aggressive form of SEF as MUC4 expression had been reported to be associated with epithelial glandular differentiation in other sarcomas. The ability to better identify and segregate the different subsets of SEF through immunohistochemistry or cytogenetics would aid clinicians to better weigh the balance between benefit and toxicity of systemic chemotherapy.

## Conclusion

In summary, in our series of patients treated at a specialist sarcoma centre, SEF is a clinically heterogeneous disease with an apparent locally and systemically infiltrative phenotype that translates to a high rate of local and metastatic recurrence. Prolonged survival may be attained in patients who can be rendered free of macroscopic disease following recurrence. Sensitivity to chemotherapy regimens widely used for STS appears to be limited. There is an urgent need for the improvement of outcomes following management of localised and advanced disease. Optimisation of currently available surgical, radiation and drug modalities should be pursued through international collaboration. Further understanding of underlying disease biology should be sought in the hope of identifying novel therapeutic vulnerabilities. As SEF is an extremely rare cancer even among sarcomas, the only feasible way of collecting a sizable patient cohort to review treatment outcomes is via collaborations with other international specialised sarcoma clinics.
